# Monitoring and evaluation of primary health care: evidence from the *Brazilian National Basic Health Units Census*

**DOI:** 10.1590/0102-311XEN160726

**Published:** 2026-11-02

**Authors:** Patty Fidelis de Almeida, Aylene Bousquat, Elaine Tomasi

**Affiliations:** 1 Instituto de Saúde Coletiva, Universidade Federal Fluminense, Niterói, Brasil.; 2 Faculdade de Saúde Pública, Universidade de São Paulo, São Paulo, Brasil.; 3 Faculdade de Medicina, Universidade Federal de Pelotas, Pelotas, Brasil.

In 2024, Brazil celebrated the 30th anniversary of the Family Health Strategy (FHS). That same year, the *Brazilian National Basic Health Units Census* (BHU Census) of the Brazilian Unified National Health System (SUS, acronym in Portuguese) was conducted. Although preceded by other significant national-level evaluation policies - such as the Improvement Access and Quality National Program of Primary Health Care (PMAQ-AB, acronym in Portuguese) - the BHU Census represented an unprecedented and innovative effort for assessing and monitoring primary health care (PHC) in Brazil. This was due to both its scope - covering all 44,938 facilities operating at the time of the survey (June to September 2024) - and its format: an online questionnaire made available through the SUS national management system, e-Gestor. 

International literature indicates that studies targeting healthcare professionals show an average response rate of 53%, with a downward trend, particularly in surveys conducted electronically ^1^. In this regard, the BHU Census can be considered, in its entire architecture, a major evaluative success - in terms of governance structure, operationalization and data collection strategies, cost, and effectiveness ^2^. The level of participation achieved by the BHU Census can also be understood not merely as the result of its design and the intense institutional mobilization involved, but - above all - as stemming from the perception of potential benefits associated with participation and the recognition of the initiative’s relevance ^3^ in giving visibility to the operating conditions of BHU. Significant participation, in turn, creates a commitment to ensure broad dissemination and feedback regarding the results. In this regard, the publication of this *Supplement* represents one of the efforts to disseminate the BHU Census results, expanding access to the evidence and analyses produced. Besides representing an innovation in strategic management within the SUS, universal participation lent the results high legitimacy and the capacity to reflect the diversity of contexts and realities of PHC in Brazil. As such, the BHU Census establishes itself as a national reference for information to support the formulation, monitoring, and improvement of public policies and evaluative studies in health.

The studies gathered in this *Supplement* explore different dimensions of PHC. It opens with a Debate - followed by commentaries - on the institutionalization of evaluation in PHC, revisiting the advances, turning points, and challenges of this process over time. The text highlights monitoring and evaluation as strategic mechanisms for social transformation, promoting equity, and strengthening the democratic principles underpinning the SUS. The eight subsequent articles explore key dimensions of Brazilian PHC in a comprehensive framework ^4^, based on the results of the BHU Census. A set of articles addresses the structural and organizational conditions of BHU, the incorporation of digital technologies, and the mechanisms for care coordination and network integration - elements that underpin PHC’s capacity to provide comprehensive and continuous care. Other studies analyze the role of PHC in responding to the population’s health needs, exploring the provision of interventions targeting chronic conditions and health promotion, and the contributions of multidisciplinary teams to expanding the scope of practice and care comprehensiveness. The *Supplement* also addresses health inequities by analyzing the provision of care to traditional peoples and communities, and the territorial differences that shape access to and the quality of care services. Finally, oral health - an essential component of the FHS - is addressed in a study examining the structural, organizational, and contextual factors associated with the expansion of services provided by oral health teams. 

The diversity of approaches covered highlights the complexity of Brazilian PHC, shedding light on how different territorial contexts, organizational arrangements, and care models influence the provision and quality of care. Taken together, the studies gathered in this *Supplement* offer not only a portrait of the PHC we currently have, but also the elements needed to reflect on the PHC we want to build. Widely recognized as the foundation of universal health systems, PHC saw its importance reinforced by the COVID-19 pandemic, which highlighted the need for well-structured services capable of coordinating care, implementing public health initiatives, and effectively responding to the needs of specific territories and populations - requiring resources commensurate with the desired scope of coverage ^5^. In this regard, strengthening PHC requires political decisions capable of reorienting priorities and ensuring sustained investment in the enhancement of care models, the workforce, infrastructure, technologies, and management strategies ^5^. The studies gathered here contribute to this debate by shedding light on challenges, potential, and pathways for building a PHC that is more effective at resolving health issues, equitable, integrated with the health care network, oriented to the needs of community, and aligned with its central role within the SUS.

Enjoy your reading!
