# Supplementary material for: Monitoring and evaluation of primary health care: evidence from the Brazilian National Basic Health Units Census
Source: Cad Saude Publica. 2026 Nov 2;42(Suppl 2):e00160726. doi: 10.1590/0102-311XEN160726 (PMC13730241; doi:10.1590/0102-311XEN160726)
Supplement: Translation

# Translation: Monitoramento e avaliação da atenção primária à saúde: evidências a partir do *Censo Nacional das Unidades Básicas de Saúde*

**DOI:** 10.1590/0102-311XPT160726

**Authors:** Patty Fidelis de Almeida, Aylene Bousquat, Elaine Tomasi

**Affiliations:** 1 Instituto de Saúde Coletiva, Universidade Federal Fluminense, Niterói, Brasil.; 2 Faculdade de Saúde Pública, Universidade de São Paulo, São Paulo, Brasil.; 3 Faculdade de Medicina, Universidade Federal de Pelotas, Pelotas, Brasil.

No ano de 2024, o Brasil comemorou os 30 anos da Estratégia Saúde da Família (ESF). Nesse mesmo ano, foi realizado o *Censo Nacional das Unidades Básicas de Saúde* (Censo das UBS) do Sistema Único de Saúde (SUS). O Censo das UBS, embora precedido por outras importantes políticas de avaliação de caráter nacional, a exemplo do Programa Nacional de Melhoria do Acesso e da Qualidade da Atenção Básica (PMAQ-AB), representou um esforço inédito e inovador no campo da avaliação e do monitoramento da atenção primária à saúde (APS) brasileira, seja pela abrangência - alcançou todos os 44.938 serviços em funcionamento no momento da realização (junho a setembro de 2024) - e pelo formato - um questionário online disponibilizado por meio de sistema nacional de gestão do SUS, e-Gestor. 

A literatura internacional aponta que estudos cujo público-alvo são profissionais de saúde apresentam uma taxa média de resposta de 53%, com tendência de redução, particularmente em pesquisas realizadas por meio eletrônico ^1^. Nessa direção, o Censo das UBS pode ser considerado, em toda a sua arquitetura, um grande êxito avaliativo - pela estrutura de governança, estratégias de operacionalização e coleta, custo e efetividade ^2^. A adesão alcançada pelo Censo das UBS pode ser compreendida, adicionalmente, não apenas como resultado do desenho e da intensa mobilização institucional, mas, sobretudo, pela percepção de possíveis benefícios envolvidos na participação e pelo reconhecimento da relevância da iniciativa ^3^ para tornar visíveis as condições de funcionamento das UBS. A participação expressiva cria, por sua vez, o compromisso de assegurar ampla divulgação e devolutiva dos resultados. Nesse sentido, a publicação deste *Suplemento* constitui um dos esforços de disseminação dos resultados do Censo das UBS, ampliando o acesso às evidências e às análises produzidas. Além de representar uma inovação para a gestão estratégica no SUS, a adesão universal conferiu aos resultados elevada legitimidade e capacidade de representar a diversidade de contextos e realidades da APS no país. Dessa forma, o Censo das UBS consolida-se como uma referência nacional de informações para subsidiar a formulação, o monitoramento e o aprimoramento de políticas públicas e estudos avaliativos em saúde.

Os estudos reunidos neste *Suplemento* exploram diferentes dimensões da APS. O *Suplemento* é aberto por um Debate, seguido por comentários, sobre a institucionalização da avaliação na APS, que revisita os avanços, as inflexões e os desafios desse processo ao longo do tempo. O texto destaca o monitoramento e a avaliação como dispositivos estratégicos para a transformação social, a promoção da equidade e o fortalecimento dos princípios democráticos que sustentam o SUS. Os oito artigos subsequentes exploram dimensões centrais da APS brasileira em uma concepção abrangente ^4^ com base nos resultados do Censo das UBS. Um conjunto de artigos aborda as condições estruturais e organizacionais das UBS, a incorporação de tecnologias digitais e os mecanismos de coordenação do cuidado e integração à rede, elementos que sustentam a capacidade da APS de ofertar atenção abrangente e contínua. Outros estudos analisam a atuação da APS na resposta às necessidades de saúde da população, explorando a oferta de ações voltadas às condições crônicas, à promoção da saúde e contribuições das equipes multiprofissionais para a ampliação do escopo de práticas e da integralidade do cuidado. O *Suplemento* também dedica atenção às inequidades em saúde, ao analisar a oferta de cuidados aos povos e comunidades tradicionais e diferenças territoriais que condicionam o acesso e a qualidade da oferta de atenção. Por fim, a saúde bucal, componente essencial da ESF, é contemplada em estudo que examina os fatores estruturais, organizacionais e contextuais associados à ampliação da oferta de ações pelas equipes de saúde bucal. 

A diversidade de abordagens contempladas evidencia a complexidade da APS brasileira, permitindo compreender como diferentes contextos territoriais, arranjos organizativos e modelos de atenção influenciam a oferta e a qualidade do cuidado. Em conjunto, os estudos reunidos neste *Suplemento* oferecem não apenas um retrato da APS que temos, mas elementos necessários para refletirmos sobre a APS que queremos construir. Amplamente reconhecida como fundamento dos sistemas universais de saúde, a APS teve sua importância reforçada pela pandemia de COVID-19, que evidenciou a necessidade de serviços bem estruturados, capazes de coordenar cuidados, desenvolver ações de saúde pública e responder de forma efetiva às necessidades dos territórios e populações, para os quais são necessários recursos compatíveis com a abrangência desejada ^5^. Nesse sentido, seu fortalecimento requer decisões políticas capazes de reorientar prioridades e assegurar investimentos sustentados na qualificação dos modelos de atenção, da força de trabalho, da infraestrutura, das tecnologias e das estratégias de gestão ^5^. Os estudos aqui reunidos contribuem para esse debate ao iluminar desafios, potencialidades e caminhos para a construção de uma APS mais resolutiva, equitativa, integrada à rede de atenção à saúde, orientada às necessidades das comunidades e compatível com a sua centralidade para o SUS.

Boa leitura!

# Translation: Seguimiento y evaluación de la atención primaria de salud: evidencia desde el *Censo Nacional de las Unidades Básicas de Salud* brasileño

**DOI:** 10.1590/0102-311XES160726

**Authors:** Patty Fidelis de Almeida, Aylene Bousquat, Elaine Tomasi

**Affiliations:** 1 Instituto de Saúde Coletiva, Universidade Federal Fluminense, Niterói, Brasil.; 2 Faculdade de Saúde Pública, Universidade de São Paulo, São Paulo, Brasil.; 3 Faculdade de Medicina, Universidade Federal de Pelotas, Pelotas, Brasil.

En el año 2024, Brasil celebró el 30º aniversario de la Estrategia de Salud Familiar (ESF). En ese mismo año se realizó el *Censo Nacional de las Unidades Básicas de Salud* (Censo de las UBS) del Sistema Único de Salud brasileño (SUS). Aunque el Censo de las UBS precede otras importantes políticas nacionales de evaluación, como el Programa de Mejora del Acceso y Calidad de la Atención Básica (PMAQ-AB, por sus siglas en portugués), representó un esfuerzo inédito e innovador en el campo de la evaluación y del seguimiento de la atención primaria de salud (APS) brasileña, ya sea por su alcance -llegó a todos los 44.938 servicios en funcionamiento al momento de su ejecución (de junio a septiembre de 2024)- o por el formato -un cuestionario en línea disponible en un sistema nacional de gestión del SUS, e-Gestor. 

La literatura internacional señala que la media de la tasa de respuesta de los estudios que se dirigen a los profesionales de la salud es del 53%, con tendencia a una disminución, particularmente, en las investigaciones realizadas electrónicamente ^1^. En este sentido, el Censo de las UBS puede considerarse, en toda su arquitectura, un gran éxito evaluativo -debido a la estructura de gobernanza, a las estrategias de puesta en práctica y recaudación, al costo y efectividad ^2^. La adhesión a que llega el Censo de las UBS también puede entenderse no solo como resultado del diseño y de la intensa movilización institucional, sino, sobre todo, por la percepción de los posibles beneficios que implica la participación y por el reconocimiento de la relevancia de la iniciativa ^3^ para visibilizar las condiciones de funcionamiento de las UBS. La participación expresiva, a su vez, crea el compromiso de garantizar una amplia difusión y retroalimentación de los resultados. En este sentido, la publicación de este *Suplemento* es uno de los esfuerzos por difundir los resultados del Censo de las UBS al ampliar el acceso a la evidencia y a los análisis producidos. Además de representar una innovación para la gestión estratégica en el SUS, la adhesión universal proporcionó resultados con alta legitimidad y capacidad para representar la diversidad de contextos y realidades de la APS en el país. Así, el Censo de las UBS se consolida como un referente nacional de información para apoyar la formulación, el seguimiento y la mejora de políticas públicas y estudios evaluativos en salud.

Los estudios recopilados en este *Suplemento* exploran diferentes dimensiones de la APS. El *Suplemento* abre con un Debate, seguido de comentarios, sobre la institucionalización de la evaluación en la APS, que revisa los avances, inflexiones y desafíos de este proceso a lo largo del tiempo. El texto destaca el seguimiento y la evaluación como dispositivos estratégicos para el cambio social, la promoción de la equidad y el fortalecimiento de los principios democráticos que sostienen el SUS. Los ocho artículos posteriores exploran las dimensiones centrales de la APS brasileña en un diseño integral ^4^ basado en los resultados del Censo de las UBS. Un conjunto de artículos aborda las condiciones estructurales y organizativas de las UBS, la incorporación de tecnologías digitales y los mecanismos para coordinar la atención y la integración en la red, elementos que respaldan la capacidad de la APS para ofrecer una atención integral y continua. Otros estudios analizan el papel de la APS para responder a las necesidades sanitarias de la población al explorar la oferta de acciones dirigidas a las condiciones crónicas, a la promoción de la salud y a los aportes de equipos multidisciplinarios para ampliar el alcance de las prácticas y la atención integral. El *Suplemento* también enfoca las desigualdades en salud al analizar la prestación de atención a los pueblos y comunidades tradicionales y las diferencias territoriales que condicionan el acceso y la calidad de la prestación asistencial. Finalmente, la salud bucal, componente esencial de la ESF, se incluye en un estudio que examina los factores estructurales, organizativos y contextuales asociados a la ampliación de la oferta de acciones por parte de los equipos de salud bucal. 

La diversidad de enfoques contemplados resalta la complejidad de la APS brasileña, lo que nos permite comprender cómo los diferentes contextos territoriales, los arreglos organizativos y los modelos de atención influyen en la oferta y la calidad de la atención. En conjunto, los estudios recopilados en este *Suplemento* ofrecen no solo una imagen de la APS que tenemos, sino también los elementos necesarios para reflexionar sobre la APS que buscamos construir. La importancia de la APS, ampliamente reconocida como la base de los sistemas universales de salud, se vio reforzada durante la pandemia de la COVID-19, que destacó la necesidad de servicios bien estructurados, capaces de coordinar la atención, desarrollar acciones de salud pública y responder eficazmente a las necesidades de los territorios y las poblaciones, para lo cual se necesitan recursos compatibles con el alcance deseado ^5^. En este sentido, su fortalecimiento requiere decisiones políticas capaces de reorientar las prioridades y asegurar inversiones sostenidas en la calificación de los modelos de atención, de la fuerza laboral, de la infraestructura, de las tecnologías y de las estrategias de gestión ^5^. Los estudios aquí reunidos contribuyen a este debate al arrojar luz sobre los desafíos, las potencialidades y las formas de construir una APS com capacidad resolutiva, equitativa, integrada a la red de atención en salud, orientada a las necesidades de las comunidades y compatible con su centralidad para el SUS.

¡Buena lectura!
